# The predictive power of phylogeny on growth rates in soil bacterial communities

**DOI:** 10.1038/s43705-023-00281-1

**Published:** 2023-07-15

**Authors:** Jeth Walkup, Chansotheary Dang, Rebecca L. Mau, Michaela Hayer, Egbert Schwartz, Bram W. Stone, Kirsten S. Hofmockel, Benjamin J. Koch, Alicia M. Purcell, Jennifer Pett-Ridge, Chao Wang, Bruce A. Hungate, Ember M. Morrissey

**Affiliations:** 1 Division of Plant and Soil Sciences, West Virginia University, Morgantown, WV 26506 USA; 2 Center for Ecosystem Science and Society (Ecoss), Northern Arizona University, Flagstaff, AZ 86011 USA; 3 Department of Biological Sciences, Northern Arizona University, Flagstaff, AZ 86011 USA; 4 Earth and Biological Sciences Directorate, Pacific Northwest National Laboratory, Richland, WA 99354 USA; 5 Department of Biological Sciences, Texas Tech University, Lubbock, TX 79409 USA; 6 Lawrence Livermore National Laboratory, Physical and Life Science Directorate, Livermore, CA USA; 7 University of California Merced, Life & Environmental Sciences Department, Merced, CA 95343 USA; 8 CAS Key Laboratory of Forest Ecology and Management, Institute of Applied Ecology, Chinese Academy of Sciences, Shenyang, LN China

**Keywords:** Microbial ecology, Soil microbiology

## Abstract

Predicting ecosystem function is critical to assess and mitigate the impacts of climate change. Quantitative predictions of microbially mediated ecosystem processes are typically uninformed by microbial biodiversity. Yet new tools allow the measurement of taxon-specific traits within natural microbial communities. There is mounting evidence of a phylogenetic signal in these traits, which may support prediction and microbiome management frameworks. We investigated phylogeny-based trait prediction using bacterial growth rates from soil communities in Arctic, boreal, temperate, and tropical ecosystems. Here we show that phylogeny predicts growth rates of soil bacteria, explaining an average of 31%, and up to 58%, of the variation within ecosystems. Despite limited overlap in community composition across these ecosystems, shared nodes in the phylogeny enabled ancestral trait reconstruction and cross-ecosystem predictions. Phylogenetic relationships could explain up to 38% (averaging 14%) of the variation in growth rates across the highly disparate ecosystems studied. Our results suggest that shared evolutionary history contributes to similarity in the relative growth rates of related bacteria in the wild, allowing phylogeny-based predictions to explain a substantial amount of the variation in taxon-specific functional traits, within and across ecosystems.

## Introduction

In soils, microorganisms participate in many ecological processes that are critically important to the maintenance of ecosystems, such as organic matter decomposition, nitrogen fixation, and nutrient immobilization [1, 2]. These ecosystem processes are determined by the aggregated traits of the individual taxa that make up microbial communities [3–5]. Unfortunately, most studies of soil bacteria characterize communities using marker gene sequencing which provides little information beyond phylogenetic community composition. To understand how community composition influences ecosystem processes we must characterize the traits of microbial taxa.

Trait-based approaches have proven useful to connect the composition of plant and animal communities with ecosystem functions for modeling [6–8]. However, the diversity of microorganisms and the difficulty associated with measuring the traits of microbial taxa in natural communities has made connecting microbial community structure and function challenging. Most environmental bacteria cannot be isolated and the few organisms that are culturable outside of their natural environments fail to adequately represent prokaryotic diversity [9, 10]. Metagenomic sequencing can provide functional ‘potential’ [11] and can be used to estimate bacterial replication rates [12]. However, genome-based indicators of functional potential often fail to predict observed traits. For instance, rRNA copy number [13] and genome size [14], are predictive of maximum growth rates in pure culture, but these traits do not correlate with growth under natural soil conditions [15]. Most molecular methods of community analysis do not distinguish active populations of microorganisms from dormant, although the latter may constitute the majority of observed taxa in a community [16], which may contribute to the disparity between growth rates in culture and natural conditions. Quantitative stable isotope probing (qSIP) enables the measurement of key microbial traits, such as relative growth rate, by measuring the amount of heavy isotope incorporation into taxon-specific DNA sequences in their natural environments [17]. Measurements of microbial function reflect the contributions active populations under specific environmental conditions, and quantifying the effects of environmental factors (such as temperature) on the traits of individual microbial taxa is an important step toward connecting microbial community composition with function. Experiments using qSIP have begun to quantify soil bacterial traits in an increasing number of ecosystems [18, 19] and in response to a variety of experimental treatments [20–23]. However, the direct measurement of bacterial traits for all taxa in all ecosystems would be an insurmountable feat. Consequently, characterization of microbial processes across all ecosystems will require methods for inferring functional processes from microbial community composition.

A phylogenetic signal in microbial functional traits (i.e. greater similarity in the traits of close relatives than expected by chance [21]) may permit trait predictions for uncharacterized taxa from phylogenies. Evolutionary processes such as rapid evolution, gene loss, and horizontal gene transfer can disrupt the phylogenetic signal in microbial functional traits. For instance, traits associated with carbohydrate metabolism in bacteria are only weakly phylogenetically clustered. In contrast, complex functional traits, such as methanogenesis and photosynthesis, that are controlled by multiple genes are more phylogenetically conserved [24, 25]. Work with simulated community and trait data suggests traits that exhibit an adequate phylogenetic signal may be amenable to phylogeny-based trait prediction [26]. Phylogeny-based trait prediction maps trait variation to a phylogenetic tree based on observed trait measurements for members of the phylogeny, then predicts trait values for ancestors and unobserved taxa based on their position within the phylogeny [27, 28]. When measured via qSIP, bacterial growth rates as well as carbon and nitrogen assimilation rates exhibit phylogenetic signals [18, 21, 28, 29], but it is unclear if the phylogenetic signal in bacterial traits is sufficient for phylogeny-based predictions.

A strong phylogenetic signal is likely to permit phylogeny-based predictions of the traits of unobserved taxa using the traits of related species measured within the same ecosystem. However, differences in phylogenetic community composition (a lack of closely related species) and trait plasticity in response to environmental variation could hamper trait prediction across ecosystems. Despite these challenges, phylogeny-based prediction across ecosystems may be possible if related organisms are present in both ecosystems and there is consistency in the estimated traits of ancestors (nodes in the phylogeny) present in both ecosystems. Additionally, challenges associated with trait plasticity may be diminished by measuring traits in experiments that manipulate environmental conditions—such as temperature, which is a principal regulator of microbial activity [4, 5]. While warming generally increases microbial activity and decomposition rates, soil carbon responses to warming temperatures remain challenging to predict. This may be because the temperature sensitivity of individual taxa varies [19], which is not currently accounted for in ecosystem models [5]. Community composition and taxon-specific temperature responses to warming were found to improve predictions of soil carbon mineralization in a controlled experiment [19, 30], phylogeny-based trait prediction could help make this possible on a larger scale. Phylogenies constructed from hundreds of thousands of genomic sequences provide a robust model of prokaryotic evolutionary history [31, 32], and widespread surveys of prokaryotic community composition provide data from diverse environments and soil conditions [33, 34]. Predicting traits from phylogeny could harness this data to estimate taxon-specific and community level function, but the accuracy of phylogeny-based trait prediction using empirical data is currently unknown.

Here we aimed to determine if the phylogenetic signal in bacterial relative growth rate is sufficient to support phylogeny-based trait prediction and examine the accuracy of phylogeny-based trait prediction within and across distinct ecosystems. Our first objective was to assess the accuracy of phylogeny-based trait predictions of bacterial relative growth rates and determine how this accuracy varied with the phylogenetic signal within ecosystems. Our second objective was to determine if there was covariation in relative growth rates for taxa and ancestral nodes shared between ecosystems that could provide a foundation for phylogeny-based trait prediction across ecosystems. Our third objective was to assess the potential for, and accuracy of, phylogeny-based prediction of bacterial relative growth rates across dissimilar ecosystems.

To address these objectives we used qSIP measurements of bacterial relative growth rates, from a previously published study of Arctic, boreal, temperate, and tropical soils (*n* = 5) [19]. As bacterial relative growth rate is highly sensitive to temperature, we tested phylogeny-based prediction of relative growth rates across a range of temperatures to gain insight into how environmental conditions may influence the utility of phylogeny-based trait predictions of bacterial activity. Bacterial phylogenies were constructed for the communities of each ecosystem incubated at each temperature and used to assess phylogenetic signals, estimate ancestral relative growth rates, and assess phylogeny-based trait prediction within and across ecosystems.

## Methods

Taxon-specific relative growth rates were quantified using quantitative stable isotope probing (qSIP) for soil microbial taxa in four ecosystems, as described by Wang et al. [19]. Briefly, soil samples were collected in August 2017 from 5 replicate plots each at the Arctic LTER site at Toolik Lake Field Station in Alaska (arctic), the SPRUCE experimental site at Marcell Experimental Forest in northern Minnesota (boreal), a mixed conifer forest site at the Hart Prairie Nature Reserve in northern Arizona (temperate), and the Sabana Field Research Station in the Luquillo Experimental Forest in Puerto Rico (tropical). Sample size was restricted due to experimental expense. All replicates were subjected to the same experimental conditions and all samples were included in analysis. Environmental and soil characteristics varied widely across the four experimental sites (Table S1). Soil samples were incubated for qSIP under uniform soil moisture conditions to allow for comparisons of growth rates across ecosystems. Soil moisture varies seasonally within all four sites, but 60% WHC is a realistically observed moisture content perennially in the Arctic and boreal ecosystems, from Autumn to Spring for the temperate ecosystem, and during the rainy season in the tropical ecosystem. Samples from each of the four sites were brought up to 60% water holding capacity with 98% ^18^O-enriched water for qSIP incubation for 5 days at 5°, 15°, 25°, and 35 °C, providing 16 different experimental groups based on ecosystem and temperature.

To assess taxon-specific relative growth rates, DNA was extracted from soil samples and separated via CsCl density gradient centrifugation and fractionation. The DNA in each fraction was purified, 16S rRNA gene copies were quantified using qPCR and the V4 region was sequenced using Illumina technology. Amplicon sequences were clustered into operational taxonomic units (OTUs or taxa) using UCLUST [35], and taxonomy was classified by aligning the most abundant sequence for each OTU with the SILVA 16S rRNA v128 gene database at 97% identity [31, 36]. Sequences were filtered for quality, excluding samples with <3500 sequence reads and excluding taxa that accounted for <0.05% of the total relative abundance in all samples [19]. Community structure of each ecosystem and temperature group varied slightly for all four ecosystems, leading to unique bacterial phylogenies. After quality filtering by OTU abundance and sequencing read depth, 888 unique OTUs with relative growth rate measurements, averaged from the five sampling reps, were included for analysis across all 16 ecosystem and temperature incubations; 205–210 OTUs in arctic soil incubations, 165–200 in boreal, 376–381 in temperate, and 257–264 in tropical. Taxon-specific relative growth rates of soil microorganisms were calculated based on the change in density following incubation in the presence of ^18^O-water, the change in density reflects the excess atom fraction of ^18^O in microbial DNA and was used to calculate a relative growth rate, expressed as a proportion per day [17, 19, 29].

Topology of the SILVA SSU gene guide tree, which is constructed by aligning full-length 16S rRNA gene sequences, was assumed to represent the phylogenetic relationships of all OTUs across the 16 experimental communities [31]. Phylogenetic analyses used the SILVA SSU guide tree pruned to include only the OTUs with observed growth rates from the qSIP experiment. To determine the influence of phylogenetic organization on bacterial growth we calculated phylogenetic signals, measured using Blomberg’s *K*, of taxon-specific relative growth rates in each ecosystem at each temperature [37]. We used residual maximum likelihood estimates of relative growth rate to estimate ancestral trait values for nodes in the phylogeny for each ecosystem and temperature combination [38]. To determine if estimated ancestral traits covary across ecosystems, we calculated the correlation coefficient (*r*) for ancestral relative growth rate estimates at nodes shared between paired ecosystems incubated at the same temperature.

We tested the potential to predict growth from phylogeny with a phylogeny-based trait prediction method implemented in *R*, based on *phyEstimate* from the package *picante* and using functions from the package *Rphylopars* [39–41]. This function uses phylogenetic tree pruning and rerooting to estimate missing trait values for individual OTUs. Briefly, for each OTU without observed trait data in a community, a phylogenetic tree is pruned to include just that unobserved OTU and the OTUs with trait data, then rerooted at the parent node of the unobserved OTU. The residual maximum likelihood estimate of the trait calculated for the root of this rearranged tree is recorded as the trait estimate for the unobserved OTU.

We first tested the accuracy of phylogeny-based estimates of bacterial growth within each community with an exclusion exercise in which trait values from 20% of the OTUs were randomly removed and then estimated. The estimated values were regressed with the observed trait values to determine estimate accuracy, quantified as the coefficient of determination (*R*^2^). We repeated this exercise 1000 times for each ecosystem and temperature combination. To determine how phylogeny-based trait prediction accuracy covaried with the strength of the associated phylogenetic signal (Objective 1) estimate accuracy values (mean *R*^2^ of estimated and observed relative growth rates) were regressed against Blomberg’s *K* measurements of phylogenetic signal across all ecosystems and temperatures.

To assess the potential for predicting traits from phylogeny across ecosystems with few shared taxa, observed trait values from each ecosystem were used to predict trait values for the other ecosystems at the same temperature. For example, relative growth rates observed at 25 °C in the Arctic ecosystem were used to predict relative growth rates at 25 °C in the Boreal, Temperate, and Tropical ecosystems. Ancestral reconstruction was performed for each ecosystem separately to permit comparison of estimates at shared nodes (Objective 2). For OTUs shared between ecosystem pairs the observed trait value measurement for the predicting ecosystem was used as the prediction. Cross-ecosystem prediction accuracy was measured as the *R*^*2*^ of estimated and observed trait values. To determine if and how phylogeny-based trait prediction accuracy across ecosystems depends upon the strength of covariation in shared estimated ancestral trait values we regressed cross-ecosystem prediction accuracy with the correlation coefficient (*r*) of shared ancestral node relative growth rate estimates for each ecosystem pair at each temperature.

## Results

**Fig. 1:**
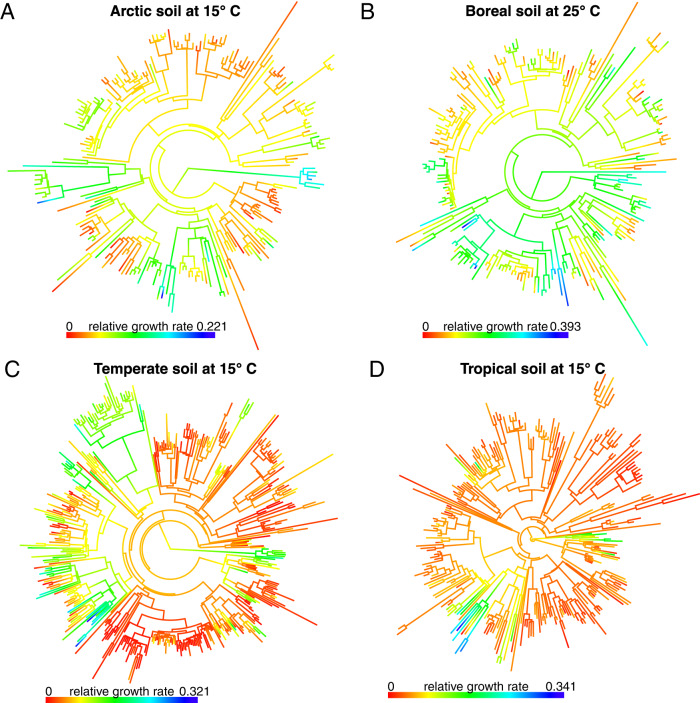
Phylogenetic trees overlayed with bacterial relative growth rates. Relative growth rates (color) are shown for taxa (tips) and estimates are provided for ancestors (nodes and branches) at 15 °C for Arctic (**A**), 25 °C for boreal (**B**), 15 °C for temperate (**C**), and 15 °C for tropical (**D**) ecosystems (note different scales).

Evolutionary history influenced bacterial relative growth rates across the Arctic, boreal, temperate, and tropical ecosystems despite differences in climate, soil organic carbon, soil nitrogen, and soil pH among the ecosystems (Supplemental Table S1). Specifically, significant phylogenetic signals were present in the relative growth rates of soil bacteria from all four ecosystems at 15°, 25°, and 35 °C, and in Arctic, boreal, and temperate ecosystems at 5 °C (Table 1). The strength of the phylogenetic signal, measured using Blomberg’s *K* values, ranged from 0.17 for the Arctic community at 35 °C to 0.66 for the temperate community at 15 °C (Table 1). The strongest phylogenetic signals were observed at 15 °C for the arctic, temperate, and tropical communities, and at 25 °C for the boreal community. Estimates of ancestral relative growth rates were calculated along the branches and nodes of phylogenetic trees for each ecosystem and temperature combination. The phylogenetic signal in relative growth rates can be visualized by coloring phylogenies according to observed values for taxa (tips), and estimated values for ancestors (branches and nodes, Fig. 1).

**Table 1:** Phylogenetic signal in the relative growth rates of soil bacteria for four ecosystems incubated at four temperatures, quantified as Blomberg’s *K* (*p-value < 0.05).

Ecosystem	Temperature			
	5	15	25	35
Arctic	0.38*	0.56*	0.44*	0.17*
Boreal	0.33*	0.30*	0.34*	0.30*
Temperate	0.47*	0.66*	0.56*	0.60*
Tropical	0.18	0.56*	0.43*	0.31*

**Fig. 2:**
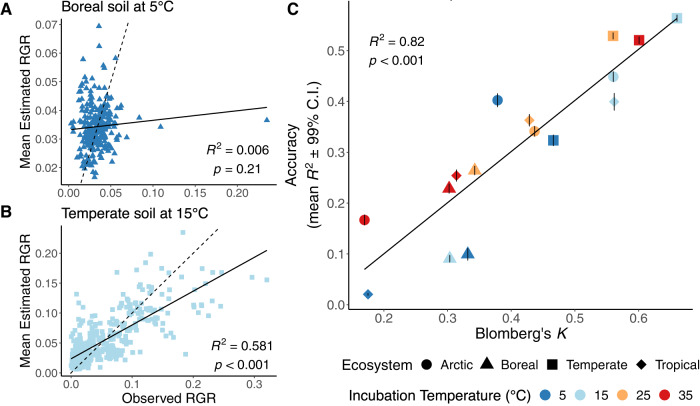
Phylogeny-based prediction accuracy (*R*^*2*^) of bacterial relative growth rates (RGRs) in relation to phylogenetic signal. Regressions (solid line) of mean observed and predicted RGR for each taxon are shown for the bacterial communities from boreal soil incubated at 5 °C (**A**), exemplifying low prediction accuracy (low *R*^*2*^), and temperate soil incubated at 15 °C (**B**), exemplifying high prediction accuracy (dash line is 1:1). Phylogeny-based trait prediction accuracy (*R*^*2*^) increased with phylogenetic signal (Blomberg’s *K*) across ecosystems and temperatures (**C**).

To determine if the phylogenetic signal in bacterial relative growth rate is sufficient to support phylogeny-based trait prediction within an ecosystem, we used a simulation exercise where trait data for 20% of the taxa (tips) were randomly excluded, predicted, and compared to observed values. Estimates from 1000 simulations for each ecosystem and temperature combination were regressed with observed values. Trait prediction accuracy ranged from *R*^2^ = 0.006 for the boreal soil community incubated at 5 °C (Fig. 2A) to *R*^2^ = 0.58 for the temperate soil incubated at 15 °C (Fig. 2B). Accuracy of the relative growth rate predictions increased linearly as the phylogenetic signal, measured as Blomberg’s *K*, increased (*R*^2^ = 0.82; Fig. 2C).

**Fig. 3:**
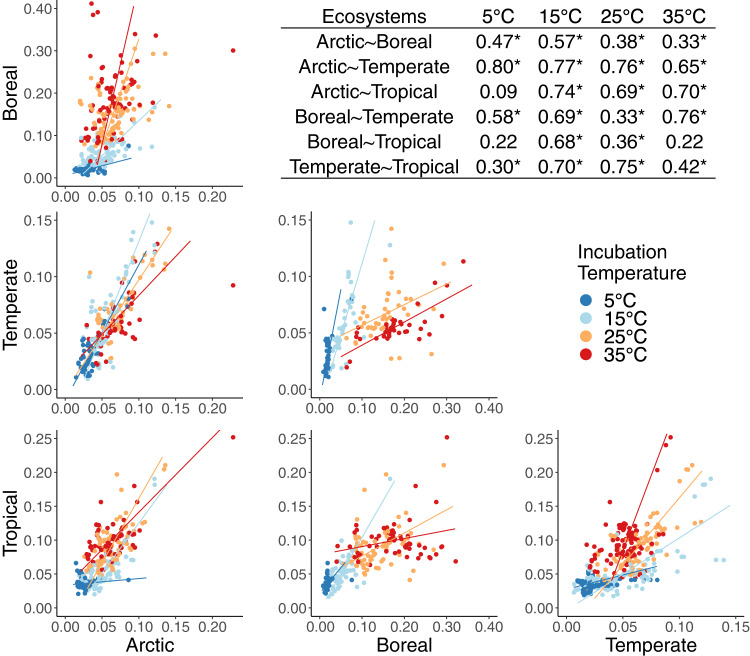
Correlations of relative growth rate estimates for ancestral nodes shared across ecosystems. Points represent estimated relative growth rates of ancestral nodes common in the phylogenies of ecosystem pairs at each incubation temperature (color). Inset table displays the Pearson correlation coefficient (*r*) for each pair (*p-value < 0.05).

**Fig. 4:**
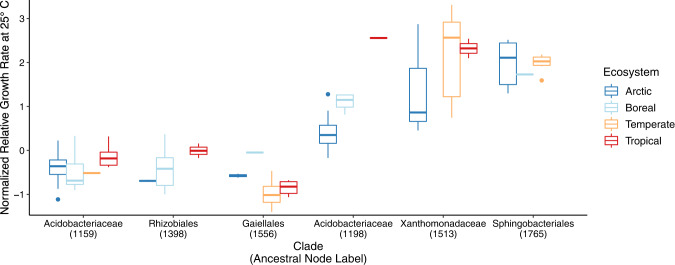
Relative growth rates (standardized Z-scores) for taxa descended from select ancestral nodes in the phylogeny. Values below and above zero reflect relative growth rates below and above the community average, respectively.

Bacterial community composition was distinct across the four ecosystems, likely due to the large climatic, geographic, and pedological differences (Supplementary Fig. S1; Table S1). Within each ecosystem community composition was similar across incubation temperatures suggesting that it did not change substantially over the incubation period (Supplementary Fig. S1). The number of observed taxa (tips) shared between any two of the ecosystems was typically fewer than 10%, and ranged from 1.5% to 26%. Despite these compositional differences at the tips of the phylogeny, the ecosystems had significant overlap deeper in the phylogeny, as evidenced by the presence of shared nodes (ancestors) common to the phylogenies derived from each ecosystem (Supplementary Fig. S2). Estimates of relative growth rates at shared ancestral nodes were linearly correlated for ecosystem pairs at most incubation temperatures (Fig. 3). The strength of these relationships, measured using Pearson’s correlation, varied across temperatures and ecosystem pairs, and ranged from weak and nonsignificant (*r* = 0.09, *p* = 0.47) to strong and highly significant (*r* = 0.80, *p* < 0.001, Fig. 3). As with the phylogenetic signals, covariation in relative growth rate estimates for shared ancestral nodes across ecosystems was strong at 15 °C. These correlations reflect similar relative growth rates in some nodes and descendant taxa across ecosystems. For example, the ancestor of Acidobacteriaceae (node 1159) and all descendant taxa had below average relative growth rates while the ancestor of Sphingobacteriales (1765) all descendant taxa had above average relative growth rates across all ecosystems (Fig. 4).

**Fig. 5:**
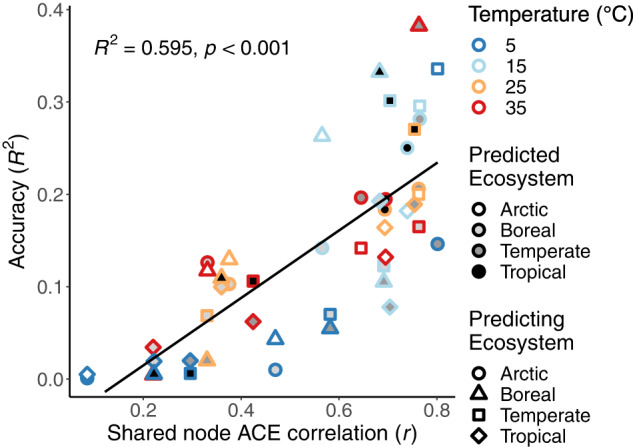
Prediction accuracy across ecosystems depends upon covariation in the estimates for shared ancestors. The phylogenetic tree and ancestral estimates from each predicting ecosystem (shape) were used to estimate relative growth rates for each predicted ecosystem (shade) at the same temperature. The accuracy (*R*^*2*^) of cross-ecosystem phylogeny-based predictions of bacterial relative growth rates increased linearly with the correlation (*r*) of ancestral estimates (ACE) for nodes shared between ecosystem (Pearson’s regression).

To test phylogeny-based trait prediction accuracy across ecosystems, we used relative growth rates from each ecosystem to predict the relative growth rates for all taxa within each of the other ecosystems at the same temperature. The predicted relative growth rates were then regressed with observed values. A substantial amount of the variation in observed trait values was explained by cross-ecosystem relative growth rate predictions in 41 out of 48 comparisons (*p* < 0.05). In general, the accuracy of predictions was related to the strength of covariation in estimated ancestral trait values at nodes shared between ecosystems (*R*^2^ = 0.59; *p* < 0.001; Fig. 5). Estimates of relative growth rates were the least accurate when soils incubated at 5 °C were compared and most accurate for comparisons among the 15 °C incubations (Fig. 5). Across ecosystems, prediction accuracy was lowest when boreal and tropical communities at 5 °C (*R*^2^ = 0.02; *p* = 0.006) were used to estimate relative growth rates of bacteria in the temperate soil, and highest when the boreal community data at 35 °C (*R*^2^ = 0.38; *p* < 0.001; Fig. 5) was used to estimate relative growth rate of bacteria in the temperate ecosystem samples.

## Discussion

Taken together our results suggest that evolutionary history imprints a phylogenetic signal on the traits of bacteria that can allow phylogeny-based predictions within and across ecosystems. Specifically, bacterial relative growth rates in soil from four ecosystems displayed phylogenetic signals sufficient for trait prediction with a meaningful level of accuracy across a range of temperatures (15°–35 °C). Consequently, phylogeny-based trait prediction may help facilitate the inclusion of biodiversity informed microbial growth and turnover rates into ecosystem models [1, 2].

In soil, bacterial growth rates are the emergent product of many genes expressed in response to spatially and temporally heterogeneous environmental conditions [15, 42, 43]. In our study, these environmental conditions, including pH, soil texture, and mean annual temperature and precipitation, varied widely between ecosystems, resulting in distinct bacterial communities with little taxonomic overlap [19]. However, there was a significant phylogenetic signal in bacterial relative growth rate in nearly all the ecosystem-temperature combinations (Table 1, Fig. 1) demonstrating the importance of evolutionary history in shaping this ecologically important trait. The strength of phylogenetic signals we observed for bacterial growth rates are consistent with previous qSIP experiments that have shown bacterial growth and assimilation of carbon and nitrogen to be evolutionarily constrained across environmental gradients [18, 28, 29]. Although the phylogenetic signal for plant and animal traits varies widely, many functional traits have comparable phylogenetic signal values to bacterial growth rates and could be suitable for comparing results of phylogeny-based analyses [37, 44]. Bacterial genes related to complex ecological functions such as nitrogen fixation, methanogenesis, and photosynthesis are often phylogenetically conserved [24] and our results suggest the genetic basis of bacterial growth rate may follow similar patterns of vertical inheritance.

The accuracy of phylogeny-based trait prediction using the traits of related species measured within the same ecosystem increased linearly with the strength of phylogenetic signal (Fig. 2). Our results are consistent with past in-silico work with simulated bacterial communities and trait data, which showed the accuracy of phylogeny-based trait predictions increases with stronger phylogenetic signal and decreases with the proportion of the community missing trait data and the mean phylogenetic distance to a taxon with a described trait [26]. Many clades, even among animals and plants, still lack sufficient observations of functional traits and consequently phylogeny is used to predict traits values [45–47]. Physiological and life strategy traits have been estimated for amphibian and mammalian species, generally with similar or higher accuracy than the best predictions of our analysis (i.e., *R*^2^ > 0.58); this could reflect the use of larger trait datasets in these studies or selection for traits that exhibit stronger phylogenetic signals [48, 49]. Phylogenetic analyses of plant and animal communities often benefit from more samples contributing to trait datasets and use phylogenies that represent a relatively narrow taxonomic clade, such as a single order, which complicates a comparison to our analysis which includes taxa from across an entire domain [45–49]. Accurate estimates of complex traits from phylogeny, such as body mass or longevity for animals and leaf area or wood density for plants, can increase the accuracy of models combining trait and environmental data to predict ecological range or threat status [45–49]. Despite greater diversity in the bacterial phylogeny and smaller trait datasets many animal and plant traits exhibit similar phylogenetic signal values to bacterial growth rates, and the accuracy of phylogeny-based predictions for these traits are similarly comparable to bacterial growth rate predictions [45–49].

The spatial distance and environmental dissimilarity of the ecosystems studied was reflected in differences in bacterial community composition [50], with relatively few taxa observed in more than one ecosystem (Supplementary Fig. S1). However, many of the ancestral nodes were shared across ecosystem pairs (Supplementary Fig. S2), and bacterial growth estimates for ancestral nodes shared between ecosystem pairs were correlated at almost every temperature. However, the strength of significant relationships, measured as Pearson’s correlation coefficient, varied drastically (Fig. 3). The relationships of ancestral character state estimates for nodes shared by communities incubated at 5 °C were the most variable and included the strongest correlation (e.g. between the Arctic and temperate soil communities), but also some of the poorest correlations (e.g. those involving the tropical ecosystem). The correlation we observed in ancestral trait values across very different ecosystems indicates a significant and consistent influence of evolutionary history on bacterial growth rates, strong enough to persist across great variation in biotic and abiotic conditions. For example, some clades had below average relative growth rates across ecosystems, these included Rhizobiales (node 1398), and Gaiellales (node 1556), while other clades, including Xanthomonadaceae (1513) and Sphingobacteriales (node 1765), had above average relative growth rates across ecosystems (Fig. 4). These consistent patterns may provide a foundation for relating phylogenetic community composition to ecosystem function across space and time.

Correlation in ancestral growth estimates between distinct ecosystems suggests phylogeny may aid in predicting functional traits, even for bacterial communities with very limited overlap in taxonomic identity. The accuracy of relative growth rate prediction, using phylogeny and trait measurements from distinct ecosystems, varied with temperature and ecosystem, and accuracy increased linearly with correlation of ancestral growth estimates at shared nodes (Fig. 5). Estimates were generally less accurate than predictions within an ecosystem (Fig. 2), which is unsurprising because trait measurements reported in these experiments are not independent from ecosystem-specific biotic and abiotic conditions. Variation in relative growth rates across the different environments and temperatures is the product of both environment and genetics, but only the latter affects trait prediction based on phylogenetic analyses. The relationship between relative growth rate prediction accuracy and consistency in ancestral relative growth rate estimates between two communities indicates that phylogeny-based trait prediction across ecosystems is only possible when phylogenetic coherence is strong enough to persist across differences in biotic and abiotic conditions. For pairings of communities with strongly correlated growth estimates across their shared ancestry up to 38% of trait variation could be explained by phylogeny alone, without accounting for environmental factors (Fig. 5). Given the great differences between the ecosystems included in this study (Supplementary Table 1), more accurate cross-ecosystem predictions may be possible for ecosystems pairs with higher biotic and abiotic similarity.

The effect of temperature on growth rate can vary across individual taxa depending on their genes, physiology, and interactions in the ecosystem. Overall, bacterial growth tended to increase with temperature, with growth in the 5 °C incubations substantially lower than in the other incubations. At 5 °C many bacteria may have been dormant with relative growth rates too low for reliable quantification, resulting in low phylogenetic signals and inconsistent correlations between ancestral relative growth rate estimates for shared nodes (Fig. 5). Thus, some experimental or environmental conditions, such as low temperatures, might prevent the application of phylogeny-based prediction of traits. At the higher incubation temperatures, the poorest ancestral growth estimate relationships were generally observed for ecosystem pairs that included the boreal soil (Fig. 5), which exhibited the highest cumulative growth rates and lowest community diversity among the four ecosystems [19]. Additionally, the strength of phylogenetic signals were generally lower in the Boreal ecosystem relative to other systems, which could be a product of decreased community diversity and more significant influence of environmental factors on relative growth rates in this ecosystem (Table 1). Our results suggest that the utility of phylogeny-based trait prediction may vary in response to biotic (e.g. diversity) and environmental (e.g. temperature) factors. Consequently, additional research may be needed to identify the circumstances under which phylogeny-based trait prediction can provide reliable estimates of microbial functional traits.

Looking forward, phylogeny-based trait prediction would benefit from trait databases that include environmental context, especially for abiotic factors, such as pH, temperature, and soil texture, that are known to explain biogeography of soil microbiomes [1, 20, 30]. In our study, phylogeny explained 38% of variance at best, and averaged just 14%, when predicting relative growth rate across ecosystems. As traits are a function of gene expression (phenotypes), a modeling effort that includes basic environmental parameters (e.g., pH and temperature) may be able to greatly improve our predictive power of phylogenetically conserved traits, like relative growth rate. Determining which microbial traits are appropriate for these methods will require substantial testing, but patterns of phylogenetic organization in both trait values and the abundance of genes associated with traits of interest indicates that phylogeny can inform ecologically relevant microbial functions [24, 51]. Modeling the interaction of environmental factors and phylogeny was beyond the scope of this project, but the data from this and similar experiments is ideal for developing such a model. As quantitative trait measurement is applied to more diverse ecosystems and processes, the increase in data will provide more reliable trait estimates. Experiments that measure bacterial traits in situ are particularly important, as results from microcosm experiments may not adequately represent ecosystem processes as they naturally occur. Increased understanding of the influence of evolutionary history on trait distribution under different environmental conditions will help determine the traits and ecosystems that would be most suitable for phylogeny-based trait prediction.

In summary, our results suggest that bacterial growth, a complex trait influenced by many heritable features, exhibits phylogenetic organization and phylogeny-based prediction can explain a substantial amount of the variation in this trait within and across ecosystems. Microbial traits such as growth rate impact how microbes transform elements within ecosystems, indeed estimates of microbial growth are often tied to rates of carbon mineralization [19, 52]. Given this, phylogeny-based predictions of microbial traits such as growth rates may help bridge the divide between phylogenetic microbial community composition and ecosystem function.

## Supplementary information

Supplementary Material

## Data Availability

Raw sequence data for this study is available in the NCBI Sequence Read Archive database under accession numbers PRJNA649787, PRJNA649546, PRJNA649571, and PRJNA649802. The relative growth rate data, and taxonomic identities for this study are available at 10.6084/m9.figshare.23145542 and the phylogenetic tree at 10.6084/m9.figshare.23057276.v.
