# Supplementary material for: The predictive power of phylogeny on growth rates in soil bacterial communities
Source: ISME Commun. 2023 Jul 15;3:73. doi: 10.1038/s43705-023-00281-1 (PMC10349831; doi:10.1038/s43705-023-00281-1)
Supplement: Supplementary file 1 — Supplementary Material [file 43705_2023_281_MOESM1_ESM.pdf]

Supplementary Material for:

The predictive power of phylogeny on growth rates in soil bacterial communities

ISME Communications, 2023

Authors:

Jeth Walkup[1], Chansotheary Dang[1], Rebecca L. Mau[2], Michaela Hayer[2], Egbert Schwartz[2,3], Bram W Stone[4], Kirsten S. Hofmockel[4], Benjamin J. Koch[2,3], Alicia M. Purcell[2,5], Jennifer Pett-Ridge [6,7], Chao Wang[8], Bruce A. Hungate[2,3], Ember M. Morrissey[1]*

[1]Division of Plant and Soil Sciences, West Virginia University, Morgantown, WV, 26506
[2]Center for Ecosystem Science and Society (Ecoss), Northern Arizona University, Flagstaff, AZ 86011
[3]Department of Biological Sciences, Northern Arizona University, Flagstaff, AZ 86011
[4]Earth and Biological Sciences Directorate, Pacific Northwest National Laboratory, Richland WA, USA 99354
[5]Department of Biological Sciences, Texas Tech University, Lubbock, TX, USA 79409
[6]Lawrence Livermore National Laboratory, Physical and Life Science Directorate, Livermore, CA, USA
[7]University of California Merced, Life & Environmental Sciences Department, Merced, CA 95343 USA
[8]CAS Key Laboratory of Forest Ecology and Management, Institute of Applied Ecology, Chinese Academy of Sciences, Shenyang, LN, China

*Corresponding Author: West Virginia University, Division of Plant and Soil Sciences, South Agricultural Sciences Building, P.O. Box 6108, Morgantown, WV, 26506, Email: ember.morrissey@mail.wvu.edu

Supplementary Table 1 Location, climate, and soil information on the ecosystems sampled for this study. Reproduced with permission from Wang et al. (2021).

	**Arctic (Alaska)**	**Boreal (Minnesota)**	**Temperate (Arizona)**	**Tropical (Puerto Rico)**
Latitude (°)	68.63 N	40.50 N	35.35N	18.3 N
Longitude (°)	149.6 W	93.48 W	111.73W	65.8 W
Elevation (m)	720	418	2620	100
Mean annual temperature (°C)	-7.4	3.3	6.6	24
Mean annual precipitation (mm)	318	768	661.2	3500
Soil organic C (mg C kg^{-1})	320.8	144.0	43.5	31.0
Soil total N (mg N kg^{-1})	12.8	6.7	3.3	2.7
Soil C/N	25.6	23	13.2	11.5
Soil pH	3.6	4.0	6.5	4.8

Wang, C., Morrissey, E. M., Mau, R. L., Hayer, M., Piñeiro, J., Mack, M. C., ... & Hungate, B. A. (2021). The temperature sensitivity of soil: microbial biodiversity, growth, and carbon mineralization. *The ISME Journal*, 1-10.

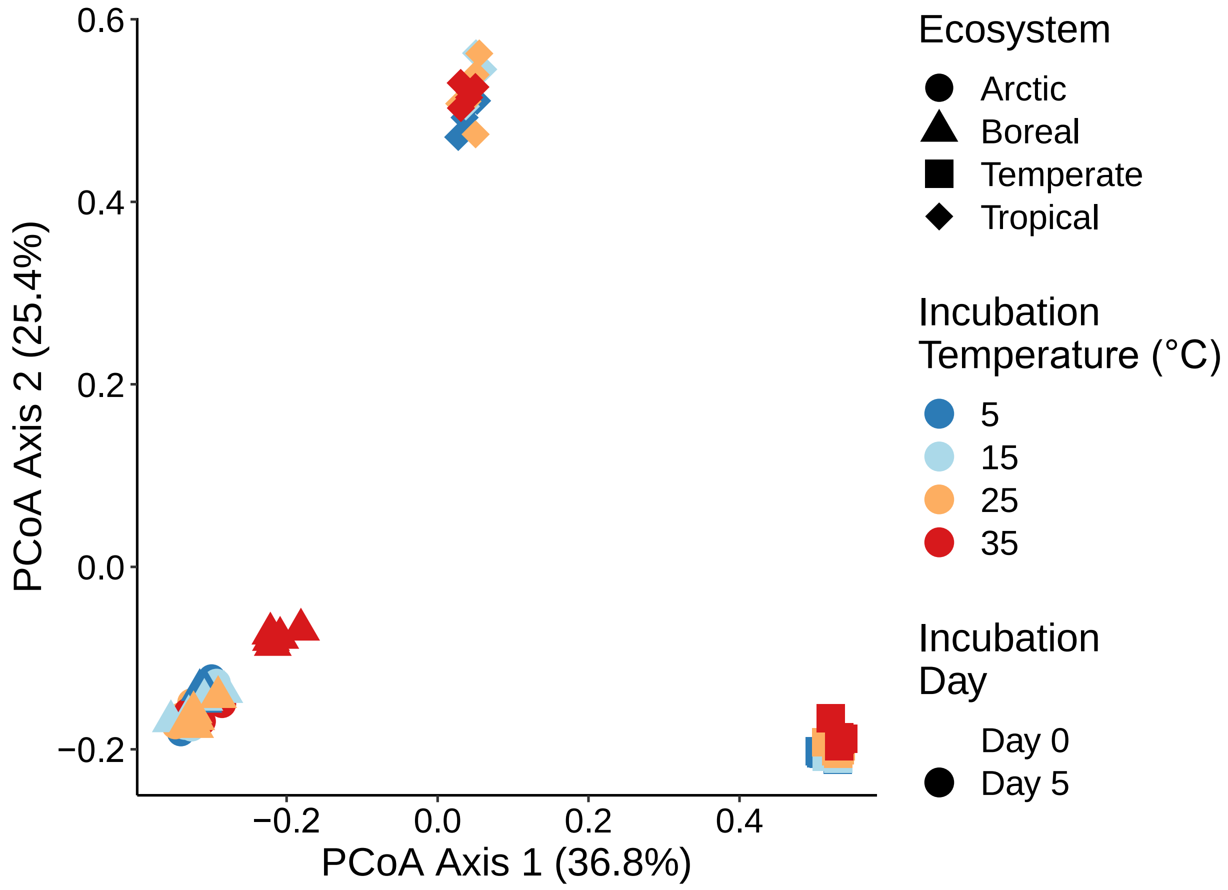

Supplemental Figure S1. Principal coordinate analysis of bacterial relative abundance in soil communities. The effects of biome, incubation day, and incubation temperature on community composition were determined with a nested 3-factor permutational multivariate analysis of variance (Y ~ Ecosystem (Incubation Day (Incubation Temperature))). All variables had a statistically significant effect on community composition (p-value < 0.001), but the explanatory power of ecosystem ($R^2 = 0.745$) was substantially stronger than incubation day ($R^2 = 0.053$) or temperature ($R^2 = 0.102$).

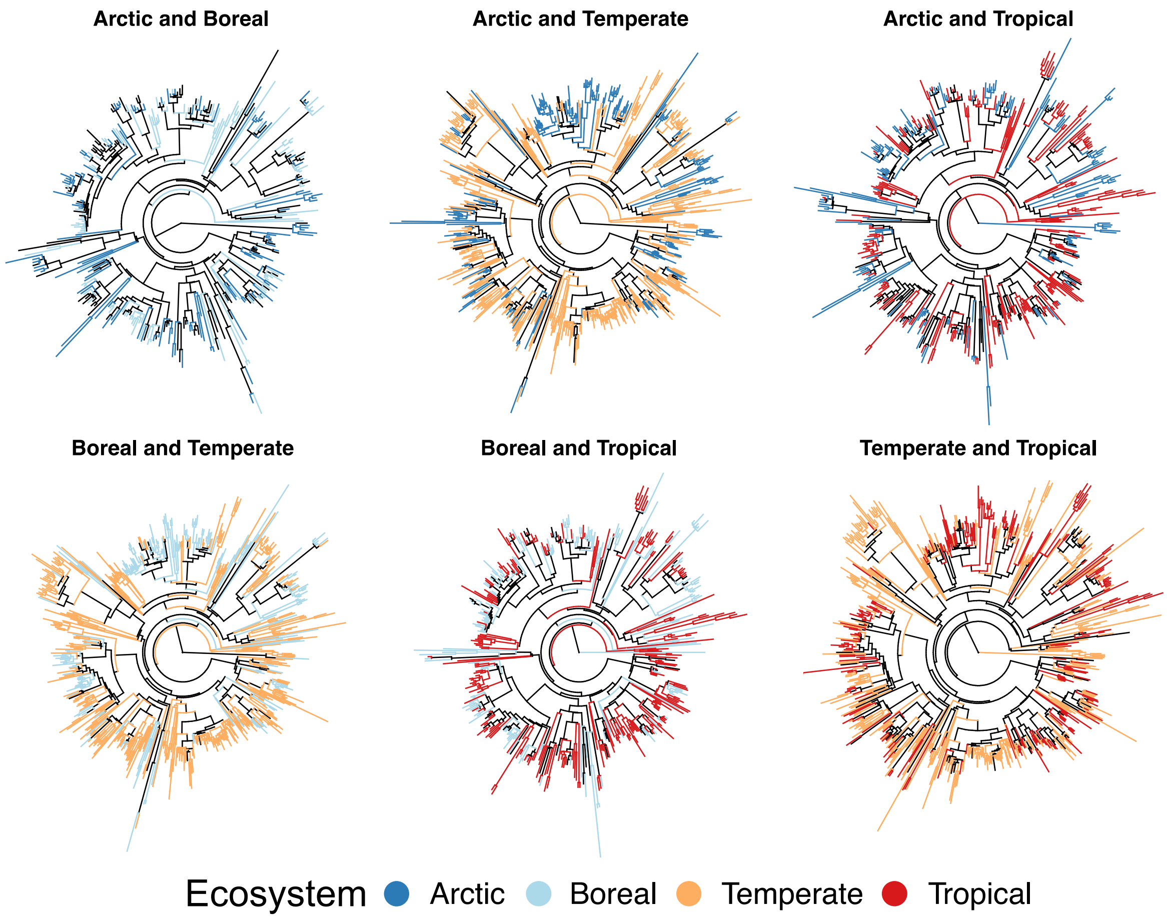

Supplemental Figure S2. Phylogenetic trees displaying the overlap (black) in phylogenetic community composition between ecosystem pairs as well as the elements unique to each ecosystem (colored by ecosystem). Overlap in the phylogeny and shared nodes (black nodes) enable cross-ecosystem phylogenetic trait prediction.
